# Genomic Analysis Reveals Novel Genes and Adaptive Mechanisms for Artificial Diet Utilization in the Silkworm Strain Guican No.5

**DOI:** 10.3390/insects15121010

**Published:** 2024-12-20

**Authors:** Lei Xin, Delong Guan, Nan Wei, Xiaoyan Zhang, Weian Deng, Xiaodong Li, Jing Song

**Affiliations:** 1 Guangxi Key Laboratory of Sericulture Ecology and Applied Intelligent Technology, Hechi University, Hechi 546399, China; xinlei@hcnu.edu.cn (L.X.); 2023660006@hcnu.edu.cn (D.G.); 13667784593@163.com (N.W.); 2022101946@hcnu.edu.cn (X.Z.); dengweian5899@163.com (W.D.); 2 Guangxi Collaborative Innovation Center of Modern Sericulture and Silk, Hechi University, Hechi 546399, China; 3 Key Laboratory of Ecology of Rare and Endangered Species and Environmental Protection, Guangxi Nomal University, Ministry of Education, Guilin 541006, China

**Keywords:** silkworm genomics, *Bombyx mori*, Guican NO.5 strain, novel gene identification, xenobiotic metabolism, artificial diet adaptation

## Abstract

The transition from traditional mulberry leaf feeding to artificial diet cultivation represents a major advancement in modern sericulture, yet the genetic mechanisms driving this adaptation remain largely unexplored. This study investigates the genomic basis of artificial diet adaptation in the silkworm strain Guican No.5 through whole-genome resequencing and transcriptome analysis. We identified 8,935,179 single-nucleotide polymorphisms (SNPs) across all chromosomes, accounting for 2.01% of the genome, with particularly high densities observed in chromosomes 23, 26, and 28. Our analysis also revealed 879 novel transcripts, many of which are involved in digestion, detoxification, and stress response pathways. Key novel genes, including three carboxylesterases, two cytochrome P450s, one heat shock protein, and one copper/zinc superoxide dismutase, exhibited varying degrees of sequence similarity to known proteins, suggesting modifications to existing genetic frameworks. Notably, one novel P450 gene displayed only 74.07% sequence identity with its closest homolog, indicating the emergence of a new protein sequence. Additionally, several key genes showed high similarity to wild silkworm (*Bombyx mandarina*) proteins, underscoring their evolutionary origins. These findings provide valuable insights into the molecular mechanisms underpinning artificial diet adaptation in silkworms and offer genomic resources to enhance artificial diet formulations and breeding programs in sericulture.

## Simple Summary

Silkworms are traditionally raised on mulberry leaves, but modern silk production increasingly uses artificial diets—manufactured food that replaces natural leaves. This shift helps overcome limitations like seasonal availability of mulberry leaves and allows year-round silk production. However, we do not fully understand how silkworms adapt to these artificial diets at the genetic level. Our study examined a special silkworm strain called Guican No.5, which grows well on artificial diet, to uncover the genetic changes that allow for this adaptation. By analyzing its complete genetic material, we discovered millions of genetic variations and hundreds of new genes that were not previously known in silkworms. Many of these new genes help in digesting artificial diet components and dealing with potentially harmful substances in the diet. We found that some of these genes came from wild silkworms but changed over time to handle artificial diet better, while others appear to be completely new. Also, we were able to identify new detoxification genes which shares low similarity with known proteins. These findings help us understand how insects adapt to new food sources and can guide the development of better artificial diets for silkworms, ultimately supporting more sustainable silk production methods that do not depend on mulberry cultivation.

## 1. Introduction

One of the most significant developments in modern sericulture has been the transition from traditional mulberry leaf feeding to artificial diet cultivation [1,2,3]. This advancement has particular importance in regions where mulberry cultivation is constrained by environmental factors or land availability [4]. Artificial diet feeding systems offer numerous advantages, including year-round production capability, reduced labor costs, and independence from seasonal mulberry leaf availability [5,6,7]. However, the genetic mechanisms underlying successful adaptation to artificial diets remain incompletely understood.

The advent of sequencing technologies has revolutionized our understanding of silkworm genetics. The 1000 Silkworm Genome Project has revealed extensive genetic diversity among different silkworm strains and provided valuable insights into their evolution and domestication history [8]. However, many locally adapted strains, particularly those developed for artificial diet cultivation, remain uncharacterized at the genomic level. Research has shown that adaptation to artificial diets involves complex genetic modifications affecting various physiological processes. Previous studies have identified several genes involved in this adaptation, particularly those related to digestion and detoxification. The detoxification system, including cytochrome P450 enzymes, plays a crucial role in processing artificial diet components [9,10]. However, the complete repertoire of genes involved in artificial diet adaptation, especially novel genes not present in reference genomes, remains to be fully characterized.

In Guangxi Province, China, the artificial diet-adapted strain “Guican No.5” has emerged as a successful example of selective breeding for artificial diet adaptation. This strain demonstrates superior growth and silk production when reared on artificial diet, making it economically significant in the region [5,11,12]. Despite its importance, Guican No.5 was not included in the 1000 Silkworm Genome Project, leaving a gap in our understanding of its genetic basis for artificial diet adaptation.

Understanding the genetic mechanisms of artificial diet adaptation has broader implications beyond sericulture [2,3,13]. Such knowledge can contribute to our understanding of insect adaptation to novel food sources, which has implications for both agricultural pest management and conservation biology. Additionally, identifying novel detoxification genes could provide insights into resistance mechanisms relevant to pest control strategies. Recent advances in genomic and transcriptomic analyses have highlighted the importance of previously uncharacterized genes in adaptive evolution [3,9,11,13]. The integration of whole-genome resequencing with transcriptome analysis has emerged as a powerful approach for identifying novel genes and regulatory mechanisms [3,13,14]. This approach is particularly valuable for understanding complex adaptations like artificial diet tolerance, which likely involves multiple genetic pathways.

The present study aims to bridge these knowledge gaps through comprehensive genomic and transcriptomic analysis of the Guican No.5 strain. By employing whole-genome resequencing and integrating it with high-depth transcriptome data, we sought to identify genetic variations and novel genes potentially contributing to artificial diet adaptation. Our findings will provide valuable insights into the molecular mechanisms underlying artificial diet adaptation and contribute genomic resources for future breeding programs. This research is particularly timely given the increasing importance of artificial diet cultivation in modern sericulture and the growing need for sustainable silk production methods. Understanding the genetic basis of artificial diet adaptation could facilitate the development of more efficiently adapted silkworm strains and improved artificial diets, ultimately contributing to the advancement of the silk industry.

## 2. Materials and Methods

### 2.1. Silkworm Strain and Sample Collection

The Guican No.5 silkworm strain was acquired from the Guangxi Sericulture Research Institute (Hechi University, Guangxi) and has been maintained exclusively on an artificial diet for over 20 generations to ensure genetic consistency and stability. Larvae were reared under controlled environmental conditions: 25  ±  1 °C temperature, 75  ±  5% relative humidity, and a 12 h light/12 h dark photoperiod.

Throughout the maintenance period, the Guican No.5 strain was not exposed to any additional chemicals, thereby eliminating potential confounding factors in subsequent analyses. For genomic analysis, genomic DNA was extracted from three fifth-instar larvae. For transcriptomic analysis, nine samples were collected, representing various developmental stages and tissues. Specifically, samples were gathered from the fifth-instar larval stage, focusing on midgut and neural tissues to capture a comprehensive overview of tissue-specific gene expression profiles. This strategic sampling approach facilitates an in-depth examination of the strain’s physiological and biochemical responses.

### 2.2. DNA Extraction and Whole-Genome Sequencing

Genomic DNA was isolated from individual fifth-instar larvae using the DNeasy Blood & Tissue Kit (Qiagen, Hilden, Germany; Cat. No. 69504) following the manufacturer’s protocol. The quality and integrity of the extracted DNA were evaluated using a NanoDrop 2000 spectrophotometer (Thermo Fisher Scientific, Waltham, MA, USA), ensuring an OD260/280 ratio between 1.8 and 2.0 and an OD260/230 ratio greater than 1.5. DNA concentration was quantified utilizing a Qubit 4.0 fluorometer ((Invitrogen, Carlsbad, CA, USA) in conjunction with the dsDNA BR Assay Kit, and DNA integrity was further confirmed by 1% agarose gel electrophoresis.

Whole-genome sequencing libraries were prepared using the NEBNext Ultra II DNA Library Prep Kit (New England BioLabs, Ipswich, MA, USA; Cat. No. E7645S) according to the manufacturer’s instructions. In brief, 1 μg of genomic DNA was fragmented to an average size of 350 bp using a Covaris S220 sonicator (Covaris Inc., Woburn, MA, USA). Post-fragmentation, end repair and A-tailing were performed, followed by ligation of sequencing adapters to the DNA fragments. The libraries underwent amplification through 8 PCR cycles and quality control was conducted using an Agilent 2100 Bioanalyzer (Agilent Technologies, Santa Clara, CA, USA). Sequencing was performed on the Illumina NovaSeq 6000 platform (Illumina, San Diego, CA, USA; Novogene, Beijing, China), generating paired-end reads of 150 bp with a target depth of 100× coverage to ensure comprehensive genome representation.

### 2.3. Bioinformatic Analysis

The newly sequenced data were filtered using fastp v0.20.0 [15] with default parameters. Clean reads were aligned to the reference *Bombyx mori* genome (SilkDB v3.0) [16] using BWA-MEM v0.7.17 [17] with default parameters. SAM files were converted to BAM format, sorted, and indexed using SAMtools v1.9 [18]. SNP calling was performed using bcftools v1.21 [18] following best practice recommendations, including base quality score recalibration and variant quality score recalibration. SNPs were filtered using the following criteria: QD < 2.0, FS > 60.0, MQ < 40.0, SOR > 3.0.

RNA-seq data were retrieved from the China National Center for Bioinformation Genome Sequence Archive under Bioproject accession PRJCA018238. These datasets originate from the Guican No.5 strain, which was subjected to chromium (Cr) stress to investigate its transcriptomic responses to heavy metal exposure [11]. A total of nine samples, representing various developmental stages and tissues, were utilized for analysis. The consensus genome of the Guican No.5 strain was newly assembled by integrating whole-genome resequencing data using bcftools v1.21 [18], providing a robust and accurate reference for subsequent transcriptomic analyses. RNA-seq reads, comprising three biological replicates from larval midgut tissues, were aligned to the consensus genome using HISAT2 v2.2.1 [19], ensuring accurate mapping across transcriptionally active regions. Novel transcripts were identified using StringTie v2.1.4 [20], defined as transcripts absent from the existing silkDB v3.0 (BmDZ.v3.6) annotation and supported by a minimum of two uniquely mapped reads. Comparative analysis against known gene models was performed using gffcompare v0.12.1 [21], further validating the uniqueness and potential functional significance of the newly identified transcripts.

Novel Gene Identification and Analysis were conducted using coding potential of novel transcripts. These sequences were assessed using both CPC2 v0.1 [22] (threshold score > 0.5) and CNCI v2.0 [23]. Transcripts predicted as coding by both tools were retained. Functional annotation was performed using the egg-nong mapper v2 [24]. Gene Ontology (GO) enrichment analysis was conducted using clusterProfiler v4.0.0 [25] in R v4.1.0 with parameters: pAdjustMethod = “BH”, pvalueCutoff = 0.05. KEGG pathway enrichment analysis was performed using the same package with similar parameters. Protein structures were predicted using AlphaFold2 [26] with default parameters. Structure comparisons were performed using TM-align. Protein properties were analyzed using ExPASy ProtParam [27]. All statistical analyses and plots generations were performed using the OmicStudio cloud platform [28]. Multiple testing corrections were implemented using the Benjamani–Hochberg method, with *p*-values < 0.05 deemed statistically significant to ensure the robustness of the findings.

## 3. Results

### 3.1. Genome-Wide SNP Calling

**Figure 1:**
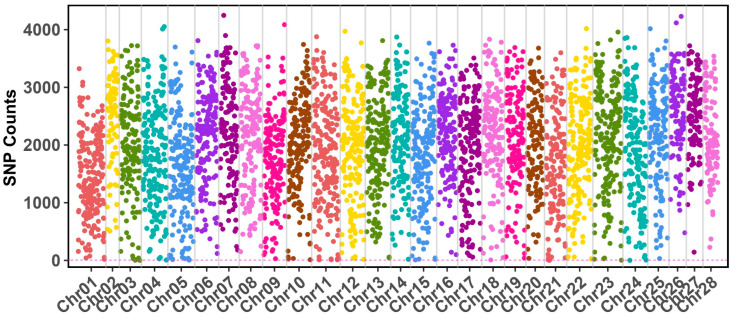
Detailed view of SNP density distribution along chromosomes of the reference genome. The *x*-axis represents chromosomal position, and the *y*-axis shows SNP counts per 100 kb window. Different colors represent individual chromosomes.

Through whole-genome sequencing, we generated a consensus genome for the Guican No. 5 strain. Compare with the reference genome of the standard strain BmDZ.v3.6.fasta retrieved from silkDB v3.0, we identified 8,935,179 SNP sites across all 28 chromosomes in the Guican No.5 strain, accounting for 2.01% of the genome (Figure 1, Table S1). Using a 100 kb sliding window analysis, we observed that SNP density varied considerably across chromosomes, ranging from approximately 100 to 4000 SNPs per window. The highest SNP counts were detected in chromosomes 23, 26, and 28, with several regions containing more than 3500 SNPs per 100 kb window. As for the chromosomal distribution pattern of SNPs, detailed analysis revealed distinct patterns of SNP distribution along each chromosome, with most chromosomes exhibiting a non-uniform distribution. A common pattern emerged where terminal regions of chromosomes showed higher SNP densities compared to their central regions. This pattern was particularly pronounced in chromosomes 4, 11, and 15, which displayed notable SNP clusters exceeding 3000 SNPs per window. In contrast, chromosomes 21 and 22 exhibited consistently lower SNP densities throughout their length, with most windows containing fewer than 2000 SNPs. Notably, chromosome 23 maintained relatively high SNP counts across its entire length, suggesting potential regions under selection during the artificial diet adaptation process.

**Figure 2:**
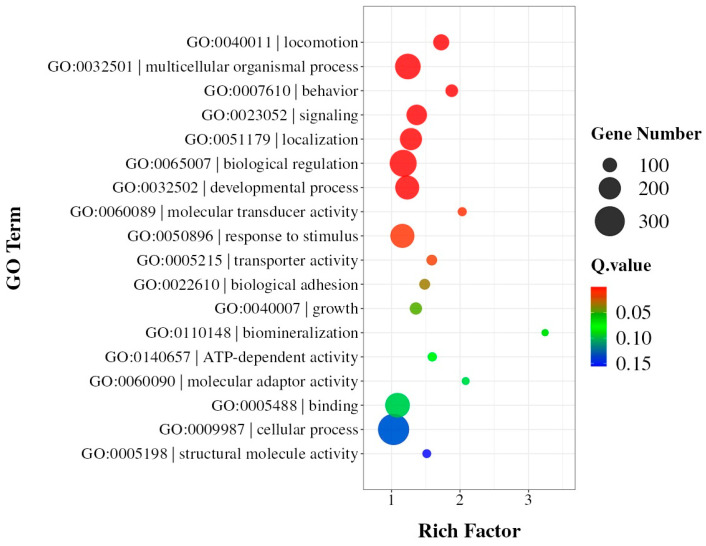
GO enrichment analysis of 451 genes containing >1000 SNPs. The size of each dot represents the number of genes in each term, and the color indicates the significance level (−log10[Q-value]). The *x*-axis shows the enrichment score.

As for the impact of SNPs on gene functions, further analysis revealed that the identified SNPs were distributed across 22,479 genes, with a total of 2,578,170 SNP sites affecting coding regions. Interestingly, the majority of genes (16,806) showed relatively low SNP density (<100 SNPs per gene), suggesting selective conservation of these genetic regions. However, 451 genes exhibited high SNP density (>1000 SNPs per gene), indicating potential regions under strong selective pressure during artificial diet adaptation. Gene Ontology (GO) enrichment analysis of these 451 highly variable genes revealed significant overrepresentation in multiple biological processes and molecular functions (Figure 2, Table S2). The most enriched GO terms included multicellular organismal process, biological regulation, and developmental process (Q-value < 0.05). Additionally, genes involved in signaling, localization, and response to stimulus were substantially enriched, suggesting their potential roles in adaptation to the artificial diet. In terms of molecular functions, transporter activity and molecular transducer activity were significantly represented, indicating possible modifications in nutrient transport and signal transduction pathways.

### 3.2. Consensus Genome Assembly and Structural Analysis

**Figure 3:**
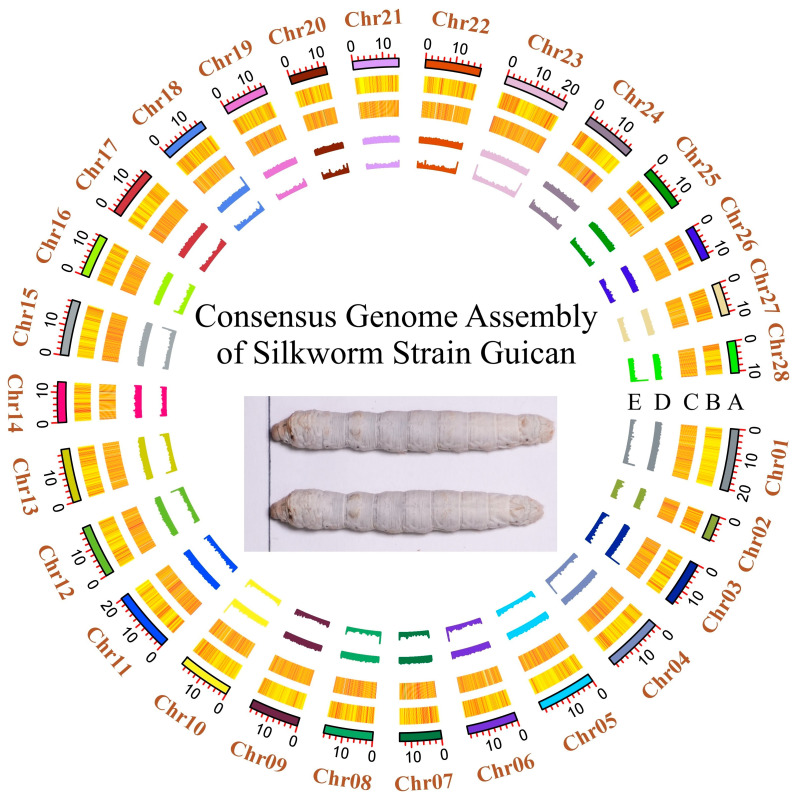
Circos plot illustrating the genomic features of the Guican No.5 genome. The plot illustrates chromosomal ideograms (track A), SNP density (track B), gene density (track C), and GC composition patterns (tracks D and E). All tracks were drawn in 100 kb sliding windows.

Based on the identified SNP polymorphisms, we generated a consensus genome assembly for the Guican No.5 strain (Figure 3). The assembly is 445.08 Mb and the N50 is 16.84 Mb. All 22,479 previously annotated silkworm genes from the reference genome (BmDZ.v3.6 from silkDBv3.0) were successfully lifted to the new assembly, indicating an overall gene structural conservation. Interestingly, we observed a slight but notable difference in GC content between the consensus (38.29%) and reference (38.33%) genomes, reflecting the massive substitution on nucleotide composition.

To identify potential novel genes unique to Guican No.5, we analyzed previously published transcriptome data (NCBI Bioproject: PRJCA018238) comprising nine samples of this strain with >6 Gb sequencing depth each. By mapping these RNA-seq reads to our newly assembled consensus genome, we initially identified 1123 potential novel transcripts in intergenic (i), unknown (u), and exonic (x) regions. To validate the uniqueness of these transcripts, we aligned them against both *B. mori* (v3.6) and *B. mandarina* reference genomes. Of the 1123 transcripts, 1122 were found in the *B. mori* reference genome, with only one non-coding transcript (located within a hypothetical protein dysbindin-like gene, GenBank: AB728505.1) being absent. In *B. mandarina*, 1049 transcripts were identified, reflecting the more distant evolutionary relationship.

**Figure 4:**
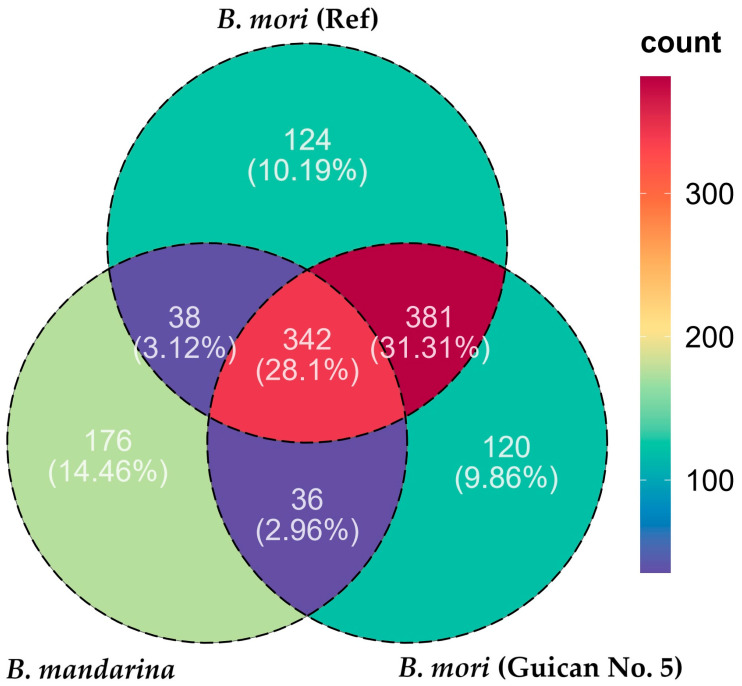
Venn diagram showing the distribution of coding domains among three silkworm genomes. Numbers in overlapping regions represent shared coding domains between genomes, while numbers in non-overlapping regions indicate unique coding domains for each genome.

Although these findings demonstrate the general conservation of our consensus genome, the observed sequence variations resulted in distinct transcriptome mapping patterns. This pattern became more pronounced when we analyzed the protein-coding potential of these transcripts across the three species (Guican No.5, *B. mori* reference, and *B. mandarina*). We identified 879, 885, and 593 coding domains, respectively, suggesting substantial variation in coding potential among these transcripts. Comparative analysis of the coding domain distribution revealed distinct patterns among the three genomes (Figure 4, Table S3). Among all identified coding domains, 342 (28.1%) were shared by all three genomes, representing the core conserved sequences. The Guican No. 5 strain shared 381 (31.31%) domains exclusively with the *B. mori* reference strain while only sharing 36 (2.96%) domains exclusively with *B. mandarina*, indicating its closer genetic relationship with the domesticated reference strain. Notably, each genome possessed a considerable number of unique coding domains: 120 (9.86%) in Guican No. 5, 124 (10.19%) in *B. mori* (reference), and 176 (14.46%) in *B. mandarina*. The higher proportion of unique coding domains in *B. mandarina* (176, 14.46%) compared to both domesticated strains suggests genomic divergence during domestication. Meanwhile, the substantial number of shared domains between Guican No. 5 and *B. mori* reference (381, 31.31%) reflects their common domestication history. The presence of unique domains in Guican No. 5 (120, 9.86%) might be associated with its specific adaptations to artificial diet and local environmental conditions.

Thus, we investigated the functions of these 120 unique domains in Guican No.5 using their Pfam annotations. After removing artifact sequences incapable of producing functional products, we identified 16 proteins with potential roles in genome plasticity and signal transduction pathway regulation. These included domains involved in signal transduction (C1_1, C2, Pkinase), chromatin regulation (Bromodomain, PHD, SET), transposition (RVT_1, Transposase_1), and developmental regulation (wnt). Notably, we identified three proteins containing retrotransposon-related domains (RVT_1) and protein kinase domains, suggesting potential roles in genome plasticity and signal transduction.

### 3.3. Identification of Key Coding Domain Sequences Related to Digestion and Detoxification

Among the unique coding domains identified in Guican No.5, we focused on genes potentially involved in digestion and detoxification processes. Through comprehensive functional annotation analysis using the EggNOG database, we were able to annotate 468 domains (53.24%) out of the 879 coding domains identified. From these annotated sequences, we identified seven Coding Domain Sequences (CDSs) related to digestion and detoxification processes: three carboxylesterase family members (MSTRG.2717, MSTRG.2723 and MSTRG.2728), two cytochrome P450 genes (MSTRG.2539 and MSTRG.3254), one heat shock protein (MSTRG.2920), and one copper/zinc superoxide dismutase (MSTRG.1993).

**Figure 5:**
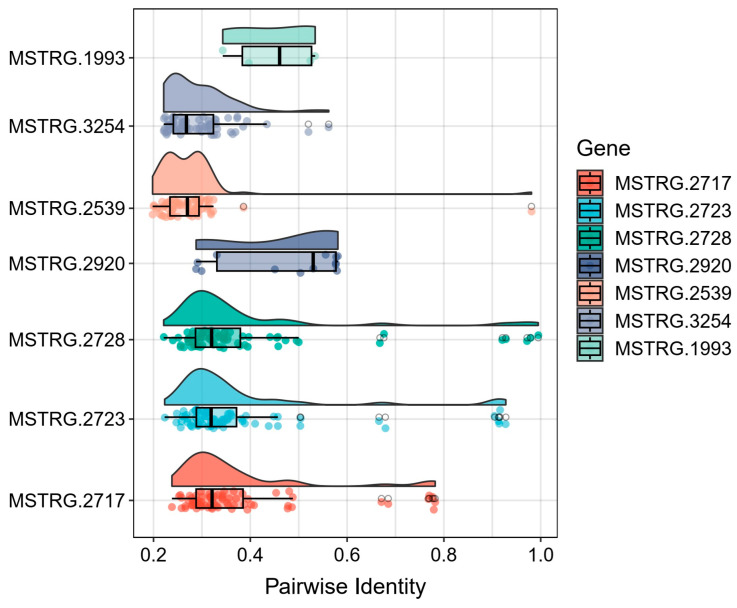
Sequence identity analysis of key novel genes against known silkworm genes. The heatmap displays pairwise sequence identity comparisons between seven newly identified genes and known silkworm genes from the reference genome. The vertical axis shows the pairwise identity values (ranging from 0 to 1), while each vertical bar in the plot represents one of the seven novel genes (MSTRG.2717, MSTRG.2723, MSTRG.2728, MSTRG.2539, MSTRG.3254, MSTRG.2920, and MSTRG.1993). Different colors are used to distinguish individual novel genes. Each point within a vertical bar indicates the sequence identity score when compared with a known silkworm gene. The distribution pattern of these points reveals the degree of conservation or divergence for each novel gene.

To evaluate the novelty of these identified genes, we conducted BLAST analysis against 22,479 known silkworm genes from the reference genome. The pairwise sequence identity distribution revealed varying degrees of similarity (Figure 5). Notably, the carboxylesterase family member MSTRG.2717 showed moderate sequence identities (30–70%), suggesting potential functional diversification while maintaining core enzymatic domains. In contrast, two other carboxylesterase candidates (MSTRG.2723 and MSTRG.2728) exhibited high sequence identity (90% and 99.5%, respectively) with known silkworm genes, indicating they likely represent new copies of existing genes.

**Figure 6:**
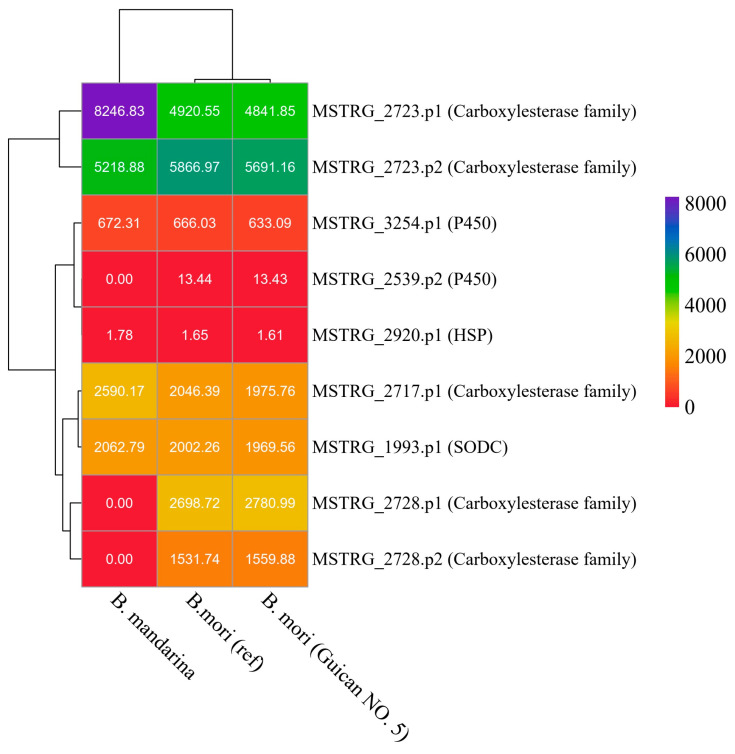
Expression analysis of novel genes across three silkworm species. Heatmap showing expression levels (TPM values) of identified novel genes in *B. mandarina*, *B. mori* reference strain, and Guican No.5. The dendrogram on the left shows hierarchical clustering of genes based on expression patterns. Gene names are shown on the right with their functional annotations in parentheses. Color scale represents expression levels from low (red) to high (purple). TPM values are indicated within each cell.

To further characterize these novel genes, we analyzed their expression levels across Guican No.5, the *B. mori* reference strain, and *B. mandarina* (Figure 6). The expression patterns revealed distinct profiles for different gene families. The carboxylesterase family members showed the highest expression levels among all analyzed genes, with MSTRG_2723 exhibiting particularly high expression in all three species (4841–8246 TPM). Notably, MSTRG_2723.p1 showed significantly higher expression in *B. mandarina* (8246.83 TPM) compared to both domesticated strains (around 4900 TPM), suggesting potential functional importance in wild populations. The cytochrome P450 genes (MSTRG_2539 and MSTRG_3254) showed moderate expression levels, with MSTRG_3254.p1 maintaining consistent expression across all three species (633–672 TPM). In contrast, MSTRG_2539.p2 showed negligible expression in *B. mandarina* but maintained low levels in both domesticated strains (approximately 13 TPM). The heat shock protein MSTRG_2920.p1 showed consistently low expression across all species (1.61–1.78 TPM), while the copper/zinc superoxide dismutase MSTRG_1993.p1 maintained moderate and stable expression levels (1969-2062 TPM). These expression patterns suggest differential regulation and potential functional specialization of these genes across wild and domesticated silkworm populations.

**Figure 7:**
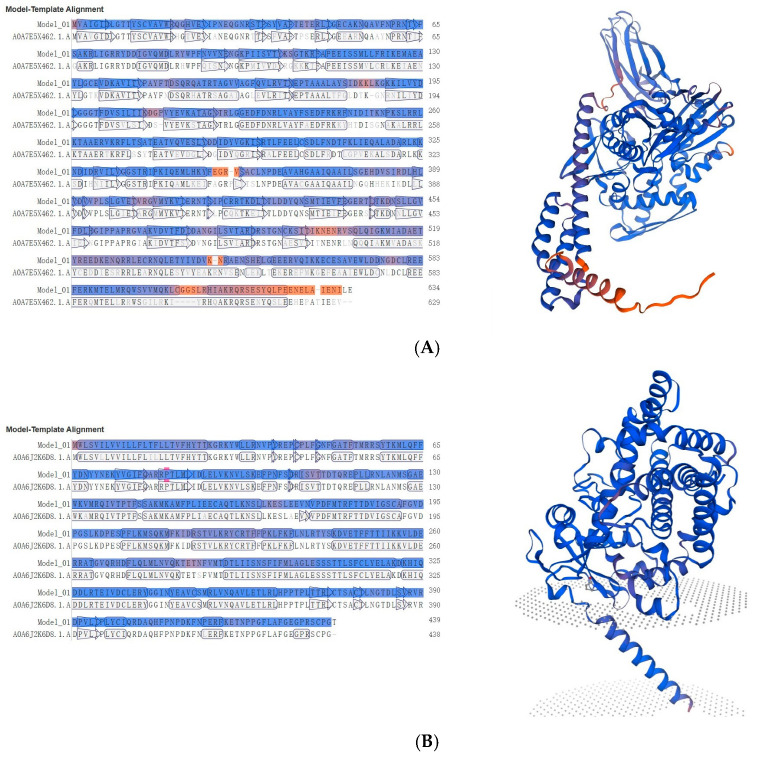

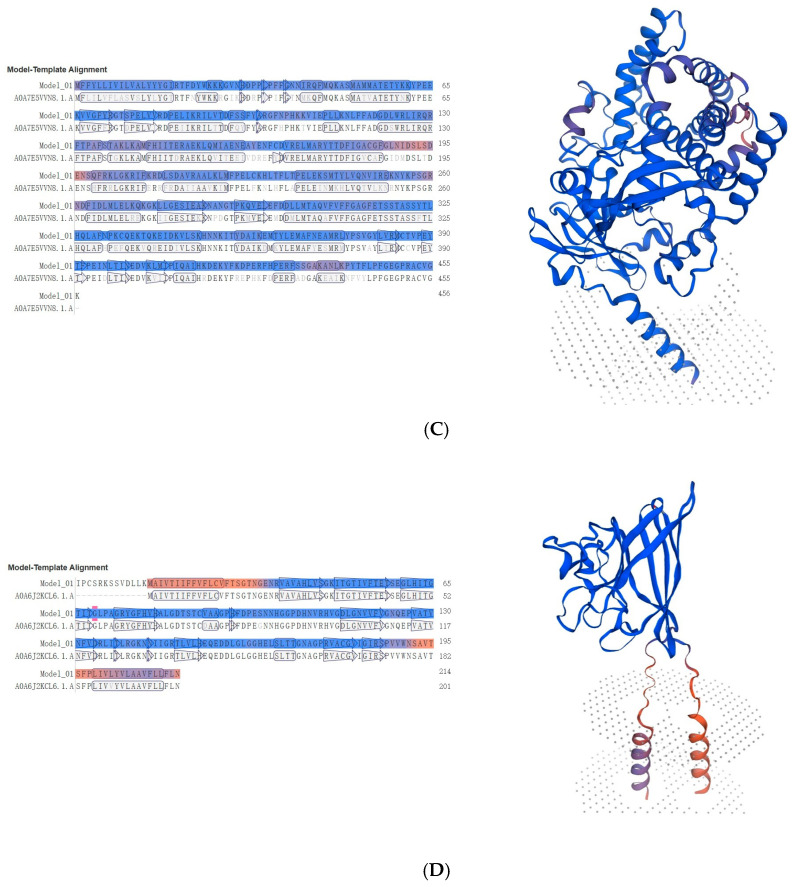
Structural analysis of four key novel digestion and detoxification proteins in Guican No.5. Left panel shows the sequence alignment, where blue regions indicate β-sheet structures and red regions represent α-helical conformations. Right panel presents the predicted three-dimensional structure model, with the β-sheet core domain shown in blue at the top and transmembrane helices in red and purple at the bottom. Gray dotted areas indicate the predicted membrane position. (**A**) MSTRG.2920 (634 aa, 71.537 kDa, pI 5.52). (**B**) MSTRG.2539 (439 aa, 50.745 kDa, pI 8.66). (**C**) MSTRG.3254 (456 aa, 52.899 kDa, pI 8.91). (**D**) MSTRG.1993 (214 aa, 22.663 kDa, pI 6.40).

Given the distinct expression patterns observed across species, we further investigated the evolutionary origins and structural features of these novel proteins that showed low similarity with existing silkworm genes through protein structure predictions and comparisons. For MSTRG.2920, which showed consistently low expression levels, its predicted structure as a heat shock protein of 634 amino acids (71.537 kDa, pI 5.52) revealed significant similarity with the AlphaFold DB model of A0A7E5X462_TRINI from *Trichoplusia ni*, showing 71.25% sequence identity (Figure 7A). This moderate similarity suggests potential functional divergence while maintaining the core heat shock protein structure. The differentially expressed cytochrome P450 proteins showed contrasting patterns of conservation. MSTRG.2539 (439 aa, 50.745 kDa, pI 8.66), despite its negligible expression in *B. mandarina*, exhibited remarkably high sequence identity (98.17%) with an unspecific monooxygenase from *Bombyx mandarina* (A0A6J2K6D8_BOMMA) (Figure 7B), suggesting strong structural conservation of this detoxification enzyme between domesticated and wild silkworms. In contrast, MSTRG.3254 (456 aa, 52.899 kDa, pI 8.91), which maintained consistent expression across species, showed only 74.07% sequence identity with its closest match, an unspecific monooxygenase from *Trichoplusia ni* (A0A7E5VVN8_TRINI) (Figure 7C), indicating substantial divergence and potentially novel functional properties. The copper/zinc superoxide dismutase MSTRG.1993 (214 aa, 22.663 kDa, pI 6.40), which displayed stable moderate expression, showed high conservation with its wild silkworm counterpart, displaying 98.51% sequence identity with the AlphaFold DB model of A0A6J2KCL6_BOMMA from *Bombyx mandarina* (Figure 7D). Notably, both MSTRG.2539 and MSTRG.1993 showed closest similarity to proteins from *Bombyx mandarina*, supporting their evolutionary origin from wild silkworm genes.

**Figure 8:**
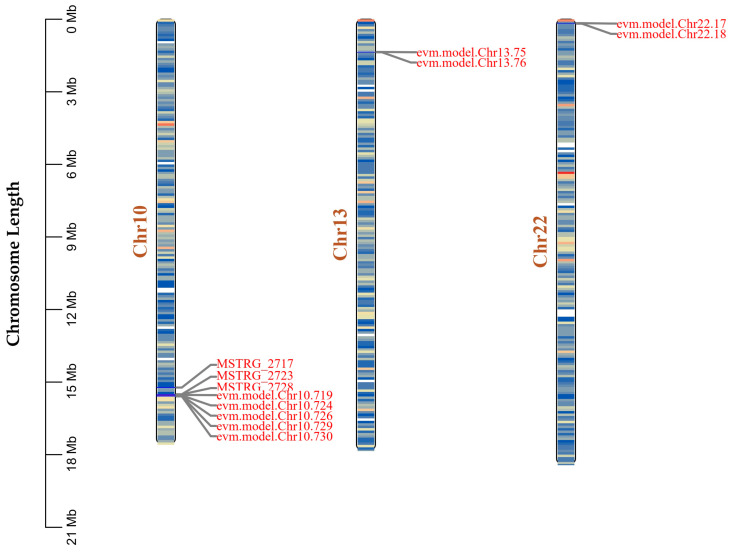
Genomic distribution of novel carboxylesterase genes and their homologs in the silkworm genome. The scale bar on the left indicates chromosome length in megabases (Mb). Gene clusters are connected by gray lines to their respective chromosomal locations.

Given the high sequence conservation of carboxylesterase family members with known *B. mori* genes revealed by our previous BLAST analysis (Figure 5), we further investigated their genomic distribution pattern. Chromosome mapping analysis showed that the three novel carboxylesterase genes (MSTRG_2717, MSTRG_2723, and MSTRG_2728) are clustered together on chromosome 10, along with five previously annotated carboxylesterase genes (evm.model.Chr10.719, 724, 726, 729, and 730) (Figure 8). This clustering pattern suggests these genes likely arose through historical tandem duplication events, a common phenomenon in the evolution of detoxification gene families.

Additionally, we identified four other previously annotated carboxylesterase genes distributed across different chromosomes: two on chromosome 13 (evm.model.Chr13.75 and 76) and two on chromosome 22 (evm.model.Chr22.17 and 18). The dispersed distribution of these genes, combined with their sequence similarities, suggests a complex evolutionary history involving both tandem and segmental duplication events. The maintenance of these duplicated genes across both wild and domesticated silkworm populations indicates their potential functional importance in silkworm biology.

## 4. Discussion

Our comprehensive genomic analysis of the artificial diet-adapted silkworm strain Guican No.5 uncovers significant genetic innovations that underpin its successful transition from traditional mulberry leaf feeding to artificial diet cultivation. The identification of nearly nine million single-nucleotide polymorphisms (SNPs), constituting 2.01% of the genome, indicates substantial genetic divergence from the reference strain. This extensive variation surpasses previously reported levels in traditional mulberry-fed strains (typically 1.2–1.5%) [8,16], highlighting the intense selection pressure imposed by artificial diet adaptation.

The non-uniform SNP distribution, particularly the elevated densities in chromosomes 23, 26, and 28, suggests these regions harbor key loci involved in adaptation. Such clustering aligns with studies on metabolic adaptation, where genomic regions underpinning environmental responses often exhibit heightened polymorphism [29,30,31]. The concentration of SNPs in the terminal regions of chromosomes, which frequently contain gene clusters related to metabolic and environmental response functions, further emphasizes their potential role in facilitating adaptation to artificial diets. The 451 highly variable genes (>1000 SNPs) identified in our study significantly overlap with pathways previously implicated in dietary adaptation, including nutrient transport and xenobiotic metabolism [14,32].

Crucially, our study identifies 879 novel transcripts with 468 protein-coding dommains, expanding the genetic repertoire associated with diet adaptation (Table S4). Among these, gene duplications and the emergence of novel gene variants—such as MSTRG.2728 and MSTRG.2723—exhibit high sequence identity with known silkworm genes yet demonstrate elevated expression levels in Guican No.5. Furthermore, the presence of retrotransposon-related domains within some of these novel genes hints at a dynamic genomic landscape, where transposable elements may drive genomic plasticity and innovation [33,34]. Moreover, our findings also reveal that several novel genes derive from wild silkworms (*Bombyx mandarina*), indicating that artificial diet adaptation may leverage pre-existing genetic diversity within wild populations [35]. This co-opting of genetic material, rather than the emergence of entirely novel genes, highlights the evolutionary plasticity of silkworms in response to anthropogenic dietary shifts [3,36]. All these results suggest that gene duplication, followed by regulatory modifications, may enhance or specialize gene functions critical for artificial diet utilization [13,14]. The presence of such closely related gene variants is consistent with the classical model of adaptive evolution, where gene duplication followed by subtle sequence modifications can lead to enhanced or specialized functions while maintaining essential catalytic activities. The remaining novel genes, particularly their enrichment in metabolic processes and detoxification pathways, provides strong evidence for their role in artificial diet utilization. Such mechanisms are consistent with the classical model of adaptive evolution, where gene duplication provides raw material for functional diversification [37,38,39].

Within the detoxification pathways, we identified novel cytochrome P450 genes (MSTRG.2539 and MSTRG.3254) exhibiting distinct evolutionary trajectories. MSTRG.2539 maintains high conservation with its wild silkworm counterpart (98.17% identity), implying a preservation of essential detoxification functions. In contrast, MSTRG.3254 displays significant divergence (74.07% identity), indicative of potential neofunctionalization tailored to metabolize specific artificial diet components. This divergence underscores the role of cytochrome P450s in adapting to novel xenobiotic environments, a phenomenon observed across various taxa [40,41]. The structural analysis of these proteins provides additional evidence for their functional specifications, with conserved catalytic domains but variable substrate-binding regions. The latter P450 gene encodes products that share relatively low similarity with known proteins, as we believe that Alphafold2 had already predicted all known proteins in human knowledge, it is suggested this MSTRG.3254 may represent a newly discovered protein sequence [26]. This finding indicated that although the silkworm genomics have extensively developed for the past two decades, there could still be novel genes that could be discovered from this fascinating insect.

The heat shock protein (MSTRG.2920) and copper/zinc superoxide dismutase (MSTRG.1993) represent different adaptive strategies. The moderate conservation of MSTRG.2920 (71.25% identity) suggests adaptation of stress response mechanisms to artificial diet conditions, while the high conservation of MSTRG.1993 (98.51% identity) indicates maintenance of critical antioxidant functions. These findings align with previous studies showing that successful dietary adaptation requires both innovation in metabolic pathways and preservation of essential protective mechanisms [42,43,44]. The discovery of multiple carboxylesterase family members, clustered on chromosome 10, suggests tandem duplication events as a pivotal mechanism in expanding detoxification capabilities. These enzymes likely facilitate the breakdown of complex artificial diet components, enhancing nutrient assimilation and mitigating potential dietary toxins [45,46,47].

Despite these advancements, our study acknowledges limitations that pave the way for future research. Functional validation of the identified novel genes through gene knockout or overexpression experiments is imperative to establish causal relationships between these genes and artificial diet adaptation. Moreover, comparative genomic analyses across additional artificial diet-adapted strains would elucidate whether the observed genetic modifications are generalizable mechanisms or unique to Guican No.5. Further investigations into the regulatory networks governing these novel genes, including tissue-specific expression patterns and dietary component-responsive elements, will deepen our understanding of the molecular orchestration behind adaptation. Exploring potential epistatic interactions among these genes could also unravel complex genetic architectures facilitating diet tolerance.

In summary, our study provides unprecedented insights into the genetic underpinnings of artificial diet adaptation in silkworms, revealing a blend of conserved essential functions and innovative gene evolution. These findings not only advance our understanding of adaptive evolution in domesticated insects but also have broader implications for optimizing sericulture practices. By leveraging the identified genetic mechanisms, it is possible to enhance artificial diet formulations and develop silkworm strains with superior performance, thereby contributing to more sustainable and resilient silk production systems.

## Data Availability

The consensus genome, called variant sites, and gene finding results for the Silkworm Strain Guican No.5 were uploaded to the figshare database under the DOI of 10.6084/m9.figshare.27639972.

## Supplementary Materials

The following supporting information can be downloaded at https://www.mdpi.com/article/10.3390/insects15121010/s1: Table S1: Variation site stats at 100 kb. Table S2: GO enrichment results for 451 highly variable genes. Table S3: The names of novel protein-coding domains across the three species (Guican No.5, *B. mori* reference, and *B. mandarina*). Table S4: The annotations of 468 novel protein-coding domains across the three species (Guican No.5, *B. mori* reference, and *B. mandarina*).

## References

[1] Shu Q., Wang Y., Gu H., Zhu Q., Liu W., Dai Y., Li F., Li B. (2023). Effects of artificial diet breeding on intestinal microbial populations at the young stage of silkworm (Bombyx mori). Arch. Insect Biochem. Physiol..

[2] Lamberti C., Gai F., Cirrincione S., Giribaldi M., Purrotti M., Manfredi M., Marengo E., Sicuro B., Saviane A., Cappellozza S. (2019). Investigation of the protein profile of silkworm (Bombyx mori) pupae reared on a well-calibrated artificial diet compared to mulberry leaf diet. PeerJ.

[3] Jiang L., Huang T., Liu Q., Zhong S., Shen D., Chen A., Zhao Q. (2023). Transcriptome analysis of anorexic and preferred silkworms (Bombyx mori) on artificial diet. Comp. Biochem. Physiol. Part D Genom. Proteom..

[4] Pan M., Jiang K., Jin Y., Mao Y., Lu W., Jiang W., Chen W. (2024). Study on the Structure and Properties of Silk Fibers Obtained from Factory All-Age Artificial Diets. Int. J. Mol. Sci..

[5] Xin L., Chen Y., Rong W., Qin Y., Li X., Guan D. (2024). Gut Microbiota Analysis in Silkworms (Bombyx mori) Provides Insights into Identifying Key Bacterials for Inclusion in Artificial Diet Formulations. Animals.

[6] Li J., Deng J., Deng X., Liu L., Zha X. (2023). Metabonomic Analysis of Silkworm Midgut Reveals Differences between the Physiological Effects of an Artificial and Mulberry Leaf Diet. Insects.

[7] Dong H.L., Zhang S.X., Chen Z.H., Tao H., Li X., Qiu J.F., Cui W.Z., Sima Y.H., Cui W.Z., Xu S.Q. (2018). Differences in gut microbiota between silkworms (Bombyx mori) reared on fresh mulberry (Morus alba var. multicaulis) leaves or an artificial diet. RSC Adv..

[8] Tong X., Han M.J., Lu K., Tai S., Liang S., Liu Y., Hu H., Shen J., Long A., Zhan C. (2022). High-resolution silkworm pan-genome provides genetic insights into artificial selection and ecological adaptation. Nat. Commun..

[9] Zhang X., Nie M., Zhao Q., Wu Y., Wang G., Xia Q. (2015). Genome-wide patterns of genetic variation among silkworms. Mol. Genet. Genom. MGG.

[10] Xin S., Zhang W. (2021). Construction and analysis of the protein-protein interaction network for the detoxification enzymes of the silkworm, Bombyx mori. Arch. Insect Biochem. Physiol..

[11] Rong W., Chen Y., Lu J., Huang S., Xin L., Guan D., Li X. (2023). Effects of Chromium Exposure on the Gene Expression of the Midgut in Silkworms, Bombyx mori. Genes.

[12] Chen Y.Z., Rong W.T., Qin Y.C., Lu L.Y., Liu J., Li M.J., Xin L., Li X.D., Guan D.L. (2023). Integrative analysis of microbiota and metabolomics in chromium-exposed silkworm (Bombyx mori) midguts based on 16S rDNA sequencing and LC/MS metabolomics. Front. Microbiol..

[13] Yin X., Zhang Y., Yu D., Li G., Wang X., Wei Y., He C., Liu Y., Li Y., Xu K. (2023). Effects of artificial diet rearing during all instars on silk secretion and gene transcription in Bombyx mori (Lepidoptera: Bombycidae). J. Econ. Entomol..

[14] Liu L., Zhao D., Wang G., He Q., Song Y., Jiang Y., Xia Q., Zhao P. (2023). Adaptive Changes in Detoxification Metabolism and Transmembrane Transport of Bombyx mori Malpighian Tubules to Artificial Diet. Int. J. Mol. Sci..

[15] Chen S., Zhou Y., Chen Y., Gu J. (2018). fastp: An ultra-fast all-in-one FASTQ preprocessor. Bioinform..

[16] Lu F., Wei Z., Luo Y., Guo H., Zhang G., Xia Q., Wang Y. (2020). SilkDB 3.0: Visualizing and exploring multiple levels of data for silkworm. Nucleic Acids Res..

[17] Jo H., Koh G. (2015). Faster single-end alignment generation utilizing multi-thread for BWA. Bio-Med. Mater. Eng..

[18] Danecek P., Bonfield J.K., Liddle J., Marshall J., Ohan V., Pollard M.O., Whitwham A., Keane T., McCarthy S.A., Davies R.M. (2021). Twelve years of SAMtools and BCFtools. GigaScience.

[19] Kim D., Paggi J.M., Park C., Bennett C., Salzberg S.L. (2019). Graph-based genome alignment and genotyping with HISAT2 and HISAT-genotype. Nat. Biotechnol..

[20] Thakur V. (2024). RNA-Seq Data Analysis for Differential Gene Expression Using HISAT2-StringTie-Ballgown Pipeline. Methods Mol. Biol..

[21] Pertea G., Pertea M. (2020). GFF Utilities: GffRead and GffCompare. F1000Research.

[22] Kang Y.J., Yang D.C., Kong L., Hou M., Meng Y.Q., Wei L., Gao G. (2017). CPC2: A fast and accurate coding potential calculator based on sequence intrinsic features. Nucleic Acids Res..

[23] Zhao J., Song X., Wang K. (2016). lncScore: Alignment-free identification of long noncoding RNA from assembled novel transcripts. Sci. Rep..

[24] Cantalapiedra C.P., Hernández-Plaza A., Letunic I., Bork P., Huerta-Cepas J. (2021). eggNOG-mapper v2: Functional Annotation, Orthology Assignments, and Domain Prediction at the Metagenomic Scale. Mol. Biol. Evol..

[25] Wu T., Hu E., Xu S., Chen M., Guo P., Dai Z., Feng T., Zhou L., Tang W., Zhan L. (2021). clusterProfiler 4.0: A universal enrichment tool for interpreting omics data. Innovation.

[26] Nunes-Alves A., Merz K. (2023). AlphaFold2 in Molecular Discovery. J. Chem. Inf. Model..

[27] Duvaud S., Gabella C., Lisacek F., Stockinger H., Ioannidis V., Durinx C. (2021). Expasy, the Swiss Bioinformatics Resource Portal, as designed by its users. Nucleic Acids Res..

[28] Lyu F., Han F., Ge C., Mao W., Chen L., Hu H., Chen G., Lang Q., Fang C. (2023). OmicStudio: A composable bioinformatics cloud platform with real-time feedback that can generate high-quality graphs for publication. iMeta.

[29] Waizumi R., Tsubota T., Jouraku A., Kuwazaki S., Yokoi K., Iizuka T., Yamamoto K., Sezutsu H. (2023). Highly accurate genome assembly of an improved high-yielding silkworm strain, Nichi01. G3: Genes Genomes Genet..

[30] Ma S.Y., Smagghe G., Xia Q.Y. (2019). Genome editing in Bombyx mori: New opportunities for silkworm functional genomics and the sericulture industry. Insect Sci..

[31] Kawamoto M., Jouraku A., Toyoda A., Yokoi K., Minakuchi Y., Katsuma S., Fujiyama A., Kiuchi T., Yamamoto K., Shimada T. (2019). High-quality genome assembly of the silkworm, Bombyx mori. Insect Biochem. Mol. Biol..

[32] Bian D., Ren Y., Ye W., Dai M., Li F., Wei J., Sun H., Li B. (2022). Evaluation of tolerance to λ-cyhalothrin and response of detoxification enzymes in silkworms reared on artificial diet. Ecotoxicol. Environ. Saf..

[33] Zhang X., Zhao M., McCarty D.R., Lisch D. (2020). Transposable elements employ distinct integration strategies with respect to transcriptional landscapes in eukaryotic genomes. Nucleic Acids Res..

[34] Li Y., Yao J., Sang H., Wang Q., Su L., Zhao X., Xia Z., Wang F., Wang K., Lou D. (2024). Pan-genome analysis highlights the role of structural variation in the evolution and environmental adaptation of Asian honeybees. Mol. Ecol. Resour..

[35] Fujimoto S., Kawamoto M., Shoji K., Suzuki Y., Katsuma S., Iwanaga M. (2020). Whole-genome sequencing and comparative transcriptome analysis of Bombyx mori nucleopolyhedrovirus La strain. Virus Genes.

[36] Li J., Chen C., Zha X. (2022). Midgut and Head Transcriptomic Analysis of Silkworms Reveals the Physiological Effects of Artificial Diets. Insects.

[37] Yuan S.F., Yue X.J., Hu W.F., Wang Y., Li Y.Z. (2023). Genome-wide analysis of lipolytic enzymes and characterization of a high-tolerant carboxylesterase from Sorangium cellulosum. Front. Microbiol..

[38] Guerrero-Cruz S., Cremers G., van Alen T.A., Op den Camp H.J.M., Jetten M.S.M., Rasigraf O., Vaksmaa A. (2018). Response of the Anaerobic Methanotroph “Candidatus Methanoperedens nitroreducens” to Oxygen Stress. Appl. Environ. Microbiol..

[39] Chertemps T., Le Goff G., Maïbèche M., Hilliou F. (2021). Detoxification gene families in Phylloxera: Endogenous functions and roles in response to the environment. Comp. Biochem. Physiol. Part D Genom. Proteom..

[40] Nauen R., Zimmer C.T., Vontas J. (2021). Heterologous expression of insect P450 enzymes that metabolize xenobiotics. Curr. Opin. Insect Sci..

[41] Lu K., Song Y., Zeng R. (2021). The role of cytochrome P450-mediated detoxification in insect adaptation to xenobiotics. Curr. Opin. Insect Sci..

[42] Liu L., Qian X., Chao M., Zhao Y., Huang J., Wang T., Sun F., Ling E., Song H. (2018). Aluminum toxicity related to SOD and expression of presenilin and CREB in Bombyx mori. Arch. Insect Biochem. Physiol..

[43] Peng Z., Hu W., Yang X., Liu Q., Shi X., Tang X., Zhao P., Xia Q. (2024). Overexpression of bond-forming active protein for efficient production of silk with structural changes and properties enhanced in silkworm. Int. J. Biol. Macromol..

[44] Kausar S., Abbas M.N., Yang L., Cui H. (2020). Biotic and abiotic stress induces the expression of Hsp70/90 organizing protein gene in silkworm, Bombyx mori. Int. J. Biol. Macromol..

[45] Zhu K., Chen Y., Chen L., Xiang H. (2022). Comparative Silk Transcriptomics Illuminates Distinctive Impact of Artificial Selection in Silkworm Modern Breeding. Insects.

[46] Wan L., Zhou A., Xiao W., Zou B., Jiang Y., Xiao J., Deng C., Zhang Y. (2021). Cytochrome P450 monooxygenase genes in the wild silkworm, Bombyx mandarina. PeerJ.

[47] Li J.Y., Cai F., Ye X.G., Liang J.S., Li J.K., Wu M.Y., Zhao D., Jiang Z.D., You Z.Y., Zhong B.X. (2017). Comparative Proteomic Analysis of Posterior Silk Glands of Wild and Domesticated Silkworms Reveals Functional Evolution during Domestication. J. Proteome Res..

